# Commissioning and clinical evaluation of a novel high‐resolution quality assurance digital detector array for SRS and SBRT

**DOI:** 10.1002/acm2.14258

**Published:** 2024-01-04

**Authors:** Yang Zhou, Yimei Liu, Meining Chen, Jianlan Fang, Liangjie Xiao, Shaomin Huang, Zhenyu Qi, Xiaowu Deng, Jun Zhang, Yinglin Peng

**Affiliations:** ^1^ State Key Laboratory of Oncology in South China, Guangdong Key Laboratory of Nasopharyngeal Carcinoma Diagnosis and Therapy, Guangdong Provincial Clinical Research Center for Cancer Sun Yat‐sen University Cancer Center Guangzhou P. R. China; ^2^ Department of Radiation Oncology, Zhuzhou Hospital Affiliated to Xiangya School of Medicine Central South University Zhuzhou P. R. China

**Keywords:** dosimetric characteristics, myQA SRS, patient‐specific quality assurance, stereotactic body radiation therapy

## Abstract

**Purpose:**

We aimed to perform the commissioning and clinical evaluation of myQA SRS detector array for patient‐specific quality assurance (PSQA) of stereotactic radiosurgery (SRS)/ stereotactic body radiotherapy (SBRT) plans.

**Methods:**

To perform the commissioning of myQA SRS, its dose linearity, dose‐rate dependence, angular dependence, and field‐size dependence were investigated. Ten SBRT plans were selected for clinical evaluation: 1) Common clinical deviations based on the original SBRT plan (Plan0), including multileaf collimator (MLC) positioning deviation and treatment positioning deviation were introduced. 2) Compared the performance of the myQA SRS and a high‐resolution EPID dosimetry system in PSQA measurement for the SBRT plans. Evaluation parameters include gamma passing rate (GPR) and distance‐to‐agreement (DTA) pass rate (DPR).

**Results:**

The dose linearity, angle dependence, and field‐size dependence of myQA SRS system exhibit excellent performance. The myQA SRS is highly sensitive in the detection of MLC deviations. The GPR of (3%/1 mm) decreases from 90.4% of the original plan to 72.7%/62.9% with an MLC outward/inward deviation of 3 mm. Additionally, when the setup error deviates by 1 mm in the X, Y, and Z directions with the GPR of (3%/1 mm) decreasing by an average of −20.9%, −25.7%, and −24.7%, respectively, and DPR (1 mm) decreasing by an average of −33.7%, −32.9%, and −29.8%. Additionally, the myQA SRS has a slightly higher GPR than EPID for PSQA, However, the difference is not statistically significant with the GPR of (3%/1 mm) of (average 90.4%% vs. 90.1%, *p* = 0.414).

**Conclusion:**

Dosimetry characteristics of the myQA SRS device meets the accuracy and sensitivity requirement of PSQA for SRS/SBRT treatment. The dose rate dependence should be adequately calibrated before its application and a more stringent GPR (3%/1 mm) evaluation criterion is suggested when it is used for SRS/SBRT QA.

## 1 INTRODUCTION

Stereotactic radiosurgery (SRS) and stereotactic body radiation therapy (SBRT) has been increasingly accepted for treatment of certain tumors, SRS/SBRT uses small‐field multiple beams and concentrate the dose distribution, with rapidly dose drop‐off (∼10% of the prescription dose per millimeter) at the outer margin to ablate the tumor while protects the organs‐at‐risk (OAR) around.^1^, ^2^ Benefited from the high biological effect of large fraction dose irradiation, it has exhibited high tumor control rates and short treatment periods for SRS/SBRT.^3^, ^4^, ^5^ However, SRS/SBRT plans generally comprise multiple small subfields, which delivery are affected by multiple factors such as the positional accuracy of the multi‐leaf collimator (MLC) and positional deviation during the actual treatment that causing largely dosimetric deviations.^6^, ^7^, ^8^ Thus, the techniques require a high degree of consistency between calculated and irradiated doses as well as positional accuracy. In other words, rigorous treatment plan dose validation must be performed prior to treatment to ensure reliable execution.

Two‐dimensional (2D) detectors, including film, ion chamber, and diode detector array are generally used in patient‐specific quality assurance (PSQA) prior to patient treatment.^9^, ^10^, ^11^, ^12^ The film detector exhibits high spatial resolution and is an ideal detector for SRS and SBRT QA. However, the film dosimetry protocol is too cumbersome, time consuming and error prone with steps of processing like scaling and scanning (single channel or multi‐channel).^13^, ^14^, ^15^ Although it has excellent detection performance, the commonly used ion chamber or diode array has relatively small number of built‐in detectors and weak in sub‐millimeter localization resolution. It is suggested that these kinds of 2D detector array need to be cross‐validated with high resolution detector like film during measurement to establish a more stringent PSQA baseline, and avoid misjudgments due to data loss in measurement of small‐field SRS/SBRT plans.^16^ With the advantages of high resolution and the potential of in vivo measurement, the amorphous‐silicon EPIDs is introduced to the application of PSQA in recent years.^17^, ^18^, ^19^, ^20^ As the flattening filter‐free (FFF) beam is used for radiotherapy especially SRS/SBRT with its high dose rates and minimized treatment time,^21^, ^22^, ^23^, ^24^ the saturation effects in EPID measurement of unfiltered high dose‐rate beams raises concerns.^24^, ^25^, ^26^, ^27^

In this paper, a novel high‐resolution digital detector array (myQA SRS array detector) was commissioned. It provides a spatial accuracy similar to film dose verification through the high resolution of digital detector arrays, making it an excellent small‐field dosimetry detector particularly suited for PSQA of SRS/SBRT plans. The objectives of this study were (1) to investigate the dosimetric characteristics of the myQA SRS detector through actual measurements, and (2) to test the detection ability and sensitivity of the myQA SRS detector for deviated SBRT delivery by introducing simulated plan deviation, compare it with the results of EPID measurement and provide reference for its widespread clinical use.

## 2 MATERIALS AND METHODS

### 2.1 Construction of the myQA SRS dose validation system

The studied dosimetry device is a high‐resolution digital detector array system (myQA SRS, IBA dosimetry, Belgium). It consists of a 2D detector array module (Detector SRS), a cylindrical phantom (diameter: 19.3 mm, buildup material: ABS, density: 1.04 g/cm^3^, RED 𝜌e,rel: 1.025) with the holder for the detector array, and the dose analysis software.(myQA Patients, version 2.15). The 2D detector array was inserted into the myQA SRS Phantom for dose validation and QA measurement, with an equivalent depth of 10 cm water for the central detector. The Detector SRS was composed of an array of monolithic solid‐state semiconductor detector with 105 000 effective points of measurement (300 × 350 pixels with 0.4 mm detector spacing), and detection area was 12 × 14 cm^2^ (Supplementary Figure 1). The measured dose was compared with the QA planned dose of the treatment planning system (TPS) in the myQA Patients software.

### 2.2 SRS detector calibration

**FIGURE 1:**
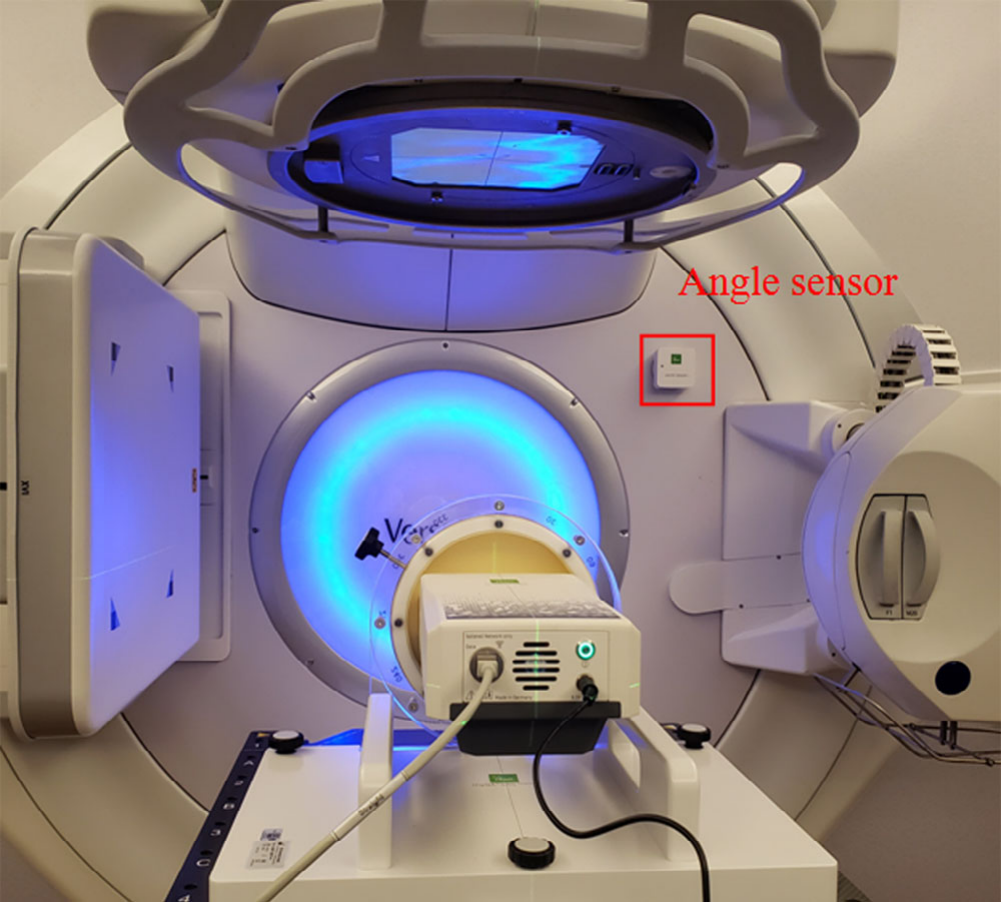
The actual measurement diagram of the experimental device. The angle sensor is marked red in the upper right corner.

A linear accelerator (Versa HD, ELEKTA) with FFF beams at high dose rate, an MLC with 80 pairs of leaves, a leaf thickness of 0.5 cm, and a maximum dose rate of 1400 MU/min were selected for the study. The output of the linear accelerator has been calibrated before the experiment. The myQA SRS measurement system was positioned on the couch, with gantry set at 0 degrees for laser positioning based on the isocenter (SAD = 100 cm). The gantry angle sensor is mounted and experimental device is connected, as shown in Figure 1.

Output calibration: Set up the myQA Phantom on the treatment tabletop with the detector‐array module inserted in and the central detector placed at the isocenter. An irradiation of 6MV FFF beam was delivered for a 10 cm × 10 cm field and 100 MU, using the linear accelerator. Dose reading of the detector array was recorded. Repeat measuring was done for three times and the average reading was taken. Replacing the detector‐array module with a calibration plug‐in, which had the same shape and density and a 0.6 cc ionization chamber in the middle, the same irradiations was repeated and measured by the ion chamber. The detector‐array was then cross‐calibrated with the results of the ionization chamber measurement.

#### 2.2.1 Gantry angle calibration

With the wireless angle detector attached onto the linear accelerator gantry, and the gantry angle set to zero, the gantry angle correction procedure was applied with the myQA SRS software system by reset the reading of the angle sensor to zero, which corrected the dependence of the sensor on the angle of irradiation field automatically.

### 2.3 Commissioning and evaluation for the myQA SRS diode‐array phantom

Before the experiment, all parameters of the accelerator such as MLC positioning accuracy, gantry positioning accuracy, isocenter accuracy, and other mechanical parameters were tested and calibrated to ensure appropriate and stable accelerator performance before absolute dose calibration. Furthermore, dose calibration of the myQA SRS dose validation system was performed.

#### 2.3.1 Dose linearity

Using the 6 MV FFF x‐ray and a dose rate of 1400 MU/min, 2, 5, 10, 20, 50, 100, 200, 500, and 1000 MU were delivered at a gantry angle of 0° and the field size of 10 cm × 10 cm. The measurements were done with the central detector of the myQA SRS placed at the isocenter and repeated three times. The average results were used for the linear correlation analysis with the delivered MU values.

#### 2.3.2 Dose‐rate dependency

A dose of 6 MV FFF x‐ray with the dose rate of 600, 800, 1000, 1200, and 1400 MU/min with the gantry angle of 0° and the field size of 10 cm × 10 cm was selected. Triply repeated measurement were done as the above set up, and the average reading were analyzed to define the consistency of the detection system at different dose rates.

#### 2.3.3 Field‐size dependency

Selecting the 6 MV FFF x‐ray and 1400 MU/min dose rate, 100 MU was delivered at a gantry angle of 0° and field size of 1.2 cm × 1.2 cm, 1.5 cm × 1.5 cm, 2 cm × 2 cm, 3 cm × 3 cm, 4 cm × 4 cm, 5 cm × 5 cm, and 10 cm × 10 cm separately. Three repeated measuring were conducted, with the central detector of the myQA SRS device placed at isocenter of 10 cm water equivalent depth. The average result of the repeated measurements was used to analyze the field‐size dependency of the detectors, and compared with those measured by a reference ionization chamber with the same condition, by replacing the myQA detector array with an ion‐chamber plug‐in. The reference measurements were run with a pinpoint ionization chamber (PTW31014, Freiburg, Germany) for all fields size smaller than 5 cm × 5 cm, and using a 0.125 cc chamber (PTW31010, Freiburg, Germany) for the fields larger than 5 cm × 5 cm, using the correction factors reported by TRS No 483.^28^

#### 2.3.4 Angular dependency

To avoid the impact of the tabletop attenuation on the dose measurement, the angular dependency was investigated with a gantry angle range of +120° to −120° in a counterclockwise direction delivering irradiation at 30° intervals. The measurements were repeated three times using the myQA SRS with the same set up as above. The averaged result of the three repetition was adopted and the measured response of different gantry angle were normalized to that of 0° gantry angle. This measurement was done with a 6 MV FFF x‐rays at 1400 MU/min dose rate and 10 cm × 10 cm field size for 100 MU irradiation.

### 2.4 Deviation planning simulation

Ten clinical SBRT plans (Plan0) implemented by linear accelerator (Versa HD, ELEKTA) were selected, including five cases of lung cancer and five cases of liver cancer. The prescription dose was 40−60 Gy in 4−10 fractions for lung cancers, and 33−57 Gy in 3−6 fractions for liver cancers. Original plan (Plan0) and the corresponding simulated deviation plans (Plan_i_) were designed and calculated in Monaco treatment planning system (Version5.1, ELEKTA). All plans used volumetric modulated arc therapy (VMAT) technique with 6 MV FFF x‐ray, and the highest dose rate is 1400 MU/min. The simulated deviations were as follows.

1. MLC deviation simulation: Based on Plan_0_, outward and inward deviations (1 , 2 , and 3 mm) of the overall MLC position were simulated, and Plan_i_ were generated, namely, Plan_MLC1_, Plan_MLC2_, and Plan_MLC3_ for outward deviation plans, and Plan_MLC1N_, Plan_MLC2N_, and Plan_MLC3N_ for inward deviation plans.
2. Setup error simulation: For Plan_0_, positioning deviations in the X, Y, and Z directions were simulated and set up with deviations of 1, 2, or 3 mm, respectively. Additionally, Plan_i_ were generated, where the deviation plans in the X direction were Plan_X1_, Plan_X2_, and Plan_X3_; the deviation plans in the Y direction were Plan_Y1_, Plan_Y2_, and Plan_Y3_; and the deviation plans in the Z direction were Plan_Z1_, Plan_Z2_, and Plan_Z3_, respectively.

The myQA SRS dose‐validation system was used for measurement and data collection of the SBRT (Plan_0_) and deviation plans (Plan_i_). Before measurement, the phantom positioning was corrected and confirmed using CBCT guidance according to the clinic protocol of SRS/SBRT in our center. After measurement, all doses were compared with the Plan_0_ QA planned doses in the myQA Patients software. Additionally, the dosimetric differences between them were analyzed, and the magnitude of the differences was evaluated using the global gamma passing rate (GPR) and distance‐to‐agreement (DTA) pass rate.^29^, ^30^ The GPR criteria were (1%/1 mm), (2%/1 mm), (2%/2 mm), (3%/1 mm), (3%/2 mm), and (3%/3 mm) with a threshold criterion of 50%. The DTA criteria were 1, 2, and 3 mm with a threshold criterion of 50%. The threshold criterion of 50% was selected to include most of the irradiated volume, focusing on the high‐dose region. The changes in GPR and DTA pass rate (DPR) were used to evaluate the ability and sensitivity of the myQA SRS to detect deviations associated with clinical execution of SBRT plans.

### 2.5 End‐to‐end testing

In the same linear accelerator (Versa HD, ELEKTA), EPID and myQA SRS were used for dosimetric verification test for 10 SBRT clinical plans (plan0) described above, respectively. The plan is designed and calculated using the Monaco TPS. The dosimetry software for EPID was EDOSE^31^ (version 5.01, Ray Dose, Guangzhou, China) and the dosimetry software for myQA SRS was mIBA Dosimetry. Record the two devices global GPRs at (3%/3 mm), (3%/2 mm), (3%/1 mm), and (2%/2 mm). For myQA verification, the same positioning protocol of clinical treatment (laser guided primely positioning and CBCT guided correction) was applied to confirm the phantom position accuracy before performing irradiation and measurement.

### 2.6 Statistics

To perform the Wilcoxon signed‐rank test on the data in each experiment, SPSS 20.0 software was used. Differences with *p* < 0.05 were considered statistically significant.

## 3 RESULTS

### 3.1 Evaluation of the myQA SRS diode‐array phantom

#### 3.1.1 Dose linearity

**FIGURE 2:**
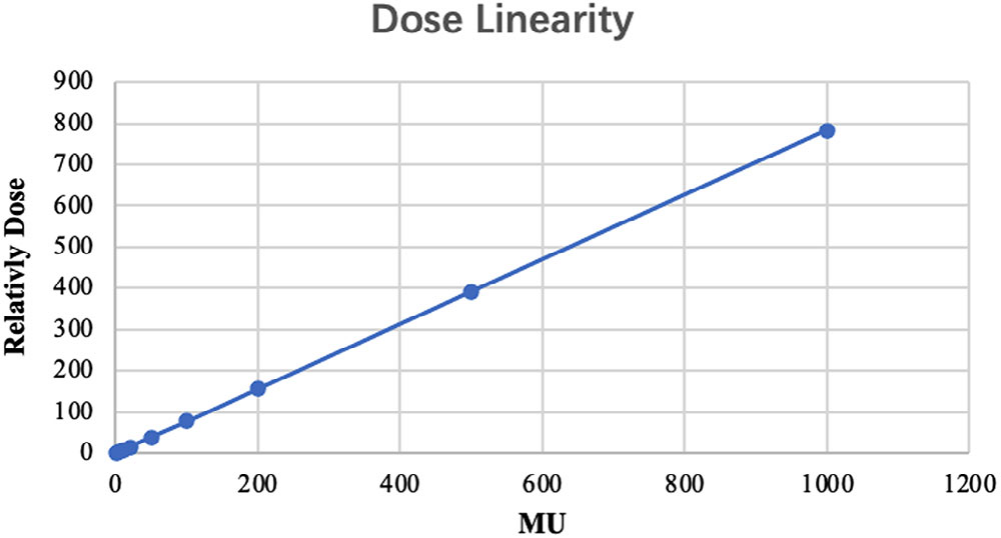
The myQA SRS detector dose linearity in the range 2−1000 MU, and the relative dose increased continuously with MU.

The myQA SRS detector exhibits good dose linearity in the range 2−1000 MU, and the relative dose increased continuously with MU. The coefficients of determination (R2) were 1.000 (Figure 2).

#### 3.1.2 Dose‐rate dependency

**FIGURE 3:**
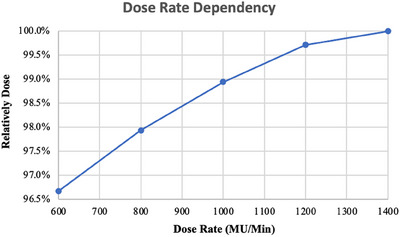
The relative dose was normalized to a measured dose rate of 1400 MU/min, and the dose rate dependency of the myQA SRS detector between 600 and 1400 MU/min.

The relative dose was normalized to a measured dose rate of 1400 MU/min, and the dose rate dependency of the myQA SRS detector was approximately 3% at different dose rates between 600 and 1400 MU/min. The relative dose increased with the dose rate (Figure 3).

#### 3.1.3 Field size dependency

**FIGURE 4:**
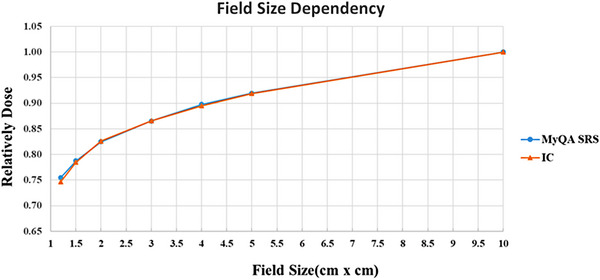
Field size output of the myQA SRS detector and IC. IC, ionization chamber.

The relative dose was normalized to a 10 cm × 10 cm field size, and the response of the myQA SRS detector to field size was compared with the ionization chamber measurements. Except for a 1.06% difference at a field size of 1.2 cm × 1.2 cm, good consistency was observed with the farmer ionization chamber at other field sizes (Figure 4).

#### 3.1.4 Gantry angle dependency

**FIGURE 5:**
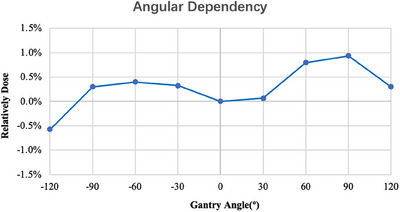
The relative dose was normalized to a measured gantry angle of 0°, and the myQA SRS detector gantry angle varied within ± 120°.

The relative dose was normalized to a measured gantry angle of 0°, and the myQA SRS detector had a small dependence of less than 1% deviation when the gantry angle varied within ± 120° (Figure 5).

### 3.2 Measurement and analysis of deviation plans

#### 3.2.1 Sensitivities of the myQA SRS detector to MLC deviation

**FIGURE 6:**
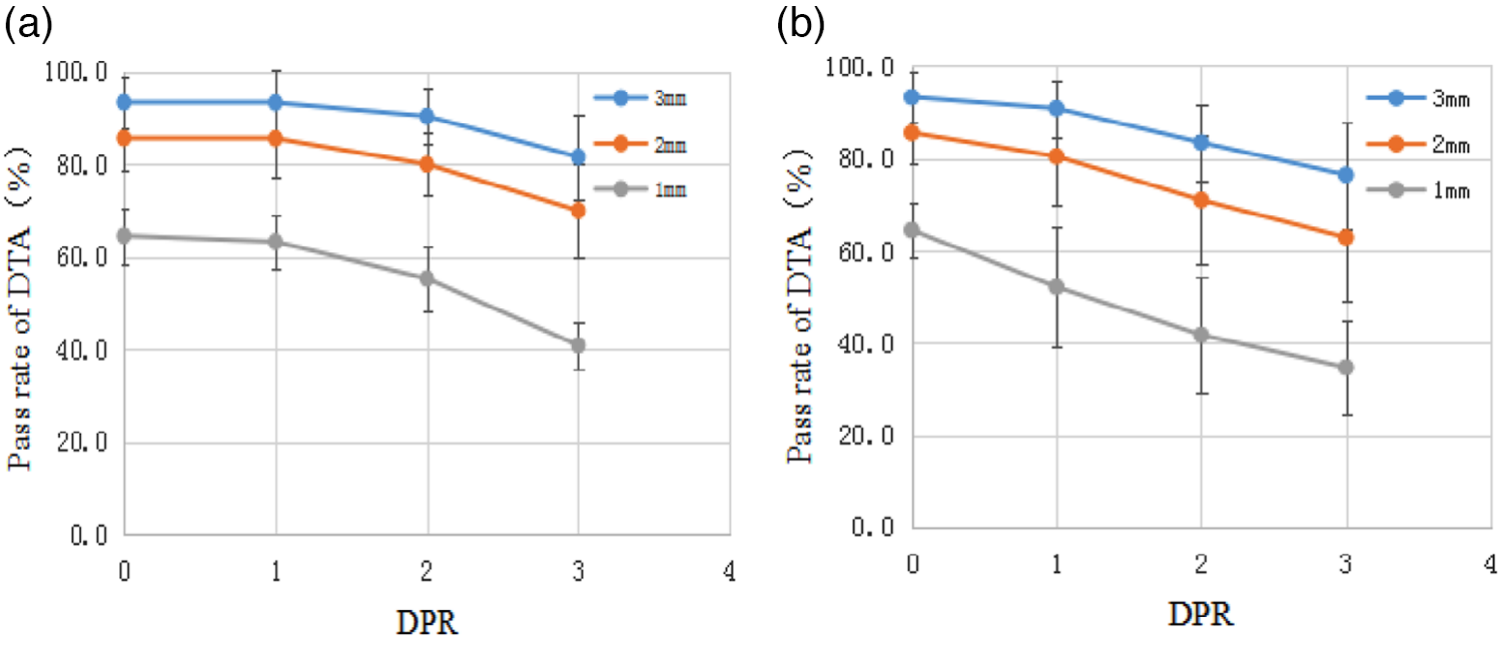
Effect of MLC positional deviation on DTA pass rate (DPR): (a) effect of outward deviation of MLC on DPR and (b) effect of inward deviation of MLC on DPR. DPR, distance‐to‐agreement pass rate.

Comparison of the dose validation results of the six MLC deviation plans (Plan_i_) showed that a 1 mm outward MLC deviation was challenging to detect using a DTA criterion greater than 1 mm. However, when a more stringent 1 mm DTA was used, the myQA SRS was very sensitive to MLC deviations. With a 1 mm outward or inward deviation of MLC, the measured GPR and DPR were decreased significantly; this decrease was more evident with increasing MLC deviation. With a 3 mm outward deviation in the MLC, the average GPR decreased from 98.3%, 90.4%, and 95.8% in the original plan (no deviation) to 88.1%, 72.7%, and 83.7% for (3%/2 mm), (3%/1 mm), and (2%/2 mm), respectively. Additionally, the 2 mm DPR decreased from 85.6% in the original plan (no deviation) to 69.9%. With a 3 mm inward deviation in the MLC, the GPR decreased to 80.1%, 62.9%, and 74.3% for (3%/2 mm), (3%/1 mm), and (2%/2 mm), respectively, and the 2 mm DPR decreased to 62.9% (Table 1, Figure 6).

**TABLE 1:** Effect of MLC deviation on gamma pass rate (%), average (minimum, maximum).

	(3%/3 mm)	(3%/2 mm)	(3%/1 mm)	(2%/2 mm)	(2%/1 mm)	(1%/1 mm)
Plan_0_	99.6 (98.3, 100.0)	98.3 (95.0, 100.0)	90.4 (81.4, 98.6)	95.8 (86.3, 99.8)	82.9 (72.4, 95.2)	69.8 (47.6, 82.9)
Plan_MLC1_	99.5 (97.0, 100.0)	98.7 (93.7, 100.0)	89.5 (68.2, 97.7)	97.5 (91.7, 99.8)	78.9 (64.3, 91.0)	69.2 (40.1, 82.6)
Plan_MLC2_	98.7 (92.6, 100.0)	96.2 (87, 100.0)	84.4 (41.9, 95.3)	94.0 (83.9, 100)	71.6 (50.0, 89.6)	67.8 (53.8, 80.5)
Plan_MLC3_	93.3 (69.3, 100.0)	88.1 (58.6, 98.4)	72.7 (25.5, 90.7)	83.7 (52.1, 95.2)	60.6 (47.6, 76.6)	53.1 (31.8, 70.3)
Plan_MLC1N_	97.9 (88.7, 100.0)	94.2 (78.4, 100.0)	81.4 (57.6, 99.6)	91.6 (73.7, 99.8)	71.4 (40.9, 93.7)	64.9 (43.6, 84.9)
Plan_MLC2N_	93.3 (77.2, 100.0)	87.0 (67.5, 100.0)	72.3 (47.0, 98.7)	82.6 (62.3, 99.5)	60.2 (36.1, 85.7)	53.6 (33.1, 75.5)
Plan_MLC3N_	87.7 (69.3, 100.0)	80.1 (57.0, 98.0)	62.9 (31.9, 93.5)	74.3 (52.1, 95.9)	51.0 (33.9, 81.6)	44.6 (25.0, 70.3)

Abbreviations: Plan_0_ was clinical SBRT plans; Plan_MLC1_, Plan_MLC2_ and Plan_MLC3_ were plans with MLC outward deviation of 1 , 2, and 3 mm, respectively; Plan_MLC1N_, Plan_MLC2N_, and Plan_MLC3N_ were plans with MLC inward deviation of 1 , 2, and 3 mm, respectively.

#### 3.2.2 Sensitivities of the myQA SRS detector to setup error

**FIGURE 7:**
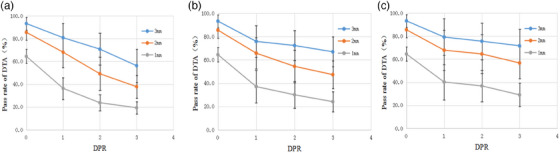
Effect of setup error on DTA pass rate (DPR): (a) effect of deviation in the X direction on DPR, (b) effect of deviation in the Y direction on DPR, and (c) effect of deviation in the Z direction on DPR. DPR, distance‐to‐agreement pass rate.

Dose validation results of nine setup error plans (Plan_i_) showed that the myQA SRS was highly sensitive to setup errors. When the set deviated by 1 mm in X, Y and Z directions, the GPR of (3%/1 mm) decreased by −20.9%, −25.7% and −24.7%, respectively, and the DPR of (1 mm) decreased by −33.7%, −32.9% and −29.8%, respectively, the decrease was more evident with increasing setup error. In the X direction, when the setup error increased from 0 to 3 mm, the GPR of (3%/2 mm), (3%/1 mm), and (2%/2 mm) decreased from averages of 98.3%, 90.4%, and 95.8% in the original plan (no deviation) to 55.3%, 41.5%, and 48.0%, respectively. Additionally, the 2 mm DPR decreased from 85.6% in the original plan (no deviation) to 37.8%. In the Y direction, when the setup error increased from 0 to 3 mm, the GPR decreased to 64.9%, 44.3%, and 57.3%, respectively, and the 2 mm DPR decreased to 47.3%. In the Z direction, when the setup error increased from 0 to 3 mm, the GPR decreased to 72.0%, 54.4%, and 66.6%, and the 2 mm DPR decreased to 56.7% (Table 2, Figure 7).

**TABLE 2:** Effect of setup error on gamma pass rate (%), average (minimum, maximum).

	(3%/3 mm)	(3%/2 mm)	(3%/1 mm)	(2%/2 mm)	(2%/1 mm)	(1%/1 mm)
Plan_0_	99.6 (98.3, 100.0)	98.3 (95.0, 100.0)	90.4 (81.4, 98.6)	95.8 (86.3, 99.9)	82.9 (72.4, 95.2)	69.8 (47.6, 82.9)
Plan_X1_	92.2 (57.1, 100.0)	85.7 (45.2, 99.4)	69.5 (30.6, 97.6)	80.3 (33.2, 98.9)	64.0 (34.7, 92.4)	46.7 (21.1, 82.3)
Plan_X2_	83.7 (35.9, 100.0)	68.2 (29.8, 96.7)	49.0 (25.3, 73.1)	61.0 (26.7, 93.3)	42.1 (23.2, 59.8)	30.6 (19.4, 49.4)
Plan_X3_	69.5 (33.5, 96.8)	55.3 (29.3, 77.8)	41.5 (22.7, 59.1)	48.0 (26.4, 61.9)	35.1 (22.7, 44.8)	25.1 (14.9, 34.8)
Plan_Y1_	89.0 (40.6, 100.0)	83.7 (29.2, 99.6)	64.7 (18.4, 93.6)	77.0 (25.1, 97.4)	59.3 (40.3, 86.4)	46.3 (12.9, 80.4)
Plan_Y2_	86.5 (40.7, 100.0)	73.6 (26.9, 95.5)	51.9 (13.2, 78.4)	65.9 (21.4, 94.2)	47.0 (28.2, 74.4)	37.2 (8.7, 68.4)
Plan_Y3_	81.1 (35.9, 99.5)	64.9 (22.7, 90.4)	44.3 (11.7, 74.2)	57.3 (18.8, 83.6)	39.3 (22.4, 62.1)	30.4 (8.0, 55.6)
Plan_Z1_	87.6 (35.3, 100.0)	81.1 (24.6, 99.6)	65.7 (13.9, 89.9)	76.5 (21.7, 98.6)	61.4 (25.8, 89.9)	49.4 (9.7, 79.2)
Plan_Z2_	84.3 (30.6, 100.0)	77.8 (21.0, 99.1)	62.7 (12.8, 89.6)	73.4 (18.9, 96.5)	57.4 (20.3, 84.0)	45.4 (8.5, 77.2)
Plan_Z3_	81.6 (30.5, 99.9)	72.0 (20.7, 91.9)	54.4 (7.9, 86.1)	66.6 (17.2, 93.8)	48.2 (15.1, 80.6)	37.3 (6.1, 71.7)

Abbreviations: Plan_0_ was clinical SBRT plans; Plan_X1_, Plan_X2_ and Plan_X3_ were plans with positioning deviations in the X axis of 1 , 2, and 3 mm, respectively; Plan_Y1_, Plan_Y2_, and Plan_Y3_ were plans with positioning deviations in the Y axis of 1 , 2, and 3 mm, respectively; Plan_Z1_, Plan_Z2_, and Plan_Z3_ were plans with positioning deviations in the Z axis of 1 , 2, and 3 mm, respectively.

### 3.3 Comparison of PSQA for SBRT

The myQA SRS detector verified the SBRT plan with higher results than the high‐resolution EPID device. However, the difference was not statistically significant with mean GPRs of (98.3% vs. 97.7%, *p* = 0.475), (90.4% vs. 90.1%, *p* = 0.414), and (95.8% vs. 95.3%, *p* = 0.610) for (3%/2 mm), (3%/1 mm), and (2%/2 mm), respectively (Table 3).

**TABLE 3:** Gamma pass rate (%) of the myQA SRS and EPID.

Patient No.	(3%/3 mm)		(3%/2 mm)		(2%/2 mm)		(3%/1 mm)	
	MyQA SRS	EPID	MyQA SRS	EPID	MyQA SRS	EPID	MyQA SRS	EPID
1	100.0	99.7	100.0	99.5	99.8	99.3	98.6	97.8
2	100.0	97.0	100.0	97.0	99.9	96.5	90.9	91.0
3	100.0	100.0	99.0	100.0	98.5	98.6	89.5	88.4
4	100.0	98.3	99.8	98.0	98.8	97.5	95.5	93.7
5	98.7	99.6	96.2	97.0	91.7	93.5	87.6	89.2
6	98.7	99.3	95.2	97.0	92.3	93.0	81.4	85.5
7	100.0	99.7	99.6	99.0	95.4	95.6	96.8	94.6
8	98.3	98.3	95.0	95.6	86.3	88.0	90.4	91.2
9	100.0	98.3	98.3	97.5	96.2	95.5	81.6	80.5
10	100.0	97.5	100.0	96.3	98.7	95.1	91.5	89.4
Average (min, max)	99.6 (98.3, 100)	98.8 (98.3, 100)	98.3 (95, 100)	97.7 (95.6, 100)	95.8 (86.3, 99.9)	95.3 (88.0, 99.3)	90.4 (81.4, 98.6)	90.1 (80.5, 97.8)
T value	−1.544		−0.715		−0.510		0.816	
*P*	0.123		0.475		0.610		0.414	

Abbreviation: EPID, electronic portal image device.

## 4 DISCUSSION

SRS/SBRT plans are characterized by high fraction doses, a steep dose drop‐off, therefore SRS/SBRT treatment requires high‐resolution detectors to measure and validate the dose and position prior to treatment. A prior study reported the QA validation of the PTW 1600SRS detector for SRS of multiple small brain metastases and revealed satisfactory validation of the treatment.^32^ The maximum resolution of the PTW 1600SRS detector was 2.5 mm in the central area (5 cm × 5 cm) and 5 mm in other measurement ranges. Thus, interpolation was required during dose analysis, leading to an extra uncertainty. The novel diode‐detector array myQA SRS, combining the advantages of higher resolution closing to film dosimetry, no interpolation is required and efficiency of digital processing, is potentially able to improve the evaluation of the of SRS/SBRT verification. James et al.^33^ compared the patient‐specific QA of four commercial QA devices for stereotypic radiotherapy plans and found that errors in MLC positioning were most reliably detected with stricter criterion for high‐resolution detectors including the myQA SRS, but not consistently detected by lower‐resolution detectors.

However, proper commissioning and evaluation to validate the devices prior to clinical use are essential in establishing confidence, minimizing errors, and understanding variations and responses in dose evaluation.^34^ In this study, we focused on the dosimetry characteristic of the myQA SRS device, tested its performance and capability in error detection for SRS/SBRT. Furthermore, we compared the results of this device with the high‐resolution EPID measurements for verification 10 clinical executed SBRT plans to validate its application.

We measured and evaluated the dose linearity, dose‐rate dependency, field‐size dependency, and gantry angle dependency of the myQA SRS detector to ensure the stability and accuracy of its dosimetry. The dose reproducibility and linearities showed sufficient performance, consistent with the test results of SRS MapCHECK detector (Sun Nuclear Corporation, USA).^35^ Many studies reported that diode arrays have dose‐rate dependency issues, including dose over‐response occurring with increasing dose rate.^36^, ^37^, ^38^, ^39^ Similarly, the myQA SRS detector exhibited a dose‐rate dependency of nearly 3% in the range 600−1400 MU/min. It would affect the accuracy of measurement if this dependency were not sufficiently corrected in measuring the dose rate‐varied irradiation like VMAT. The myQA SRS system has integrated the dose rate dependency calibration function in its software system, which can monitor the dose rate and correct the measurement instantly, if this dependency was pre‐investigated and input to the system. That means this dependency should be carefully inspected during the system commissioning. In addition, the myQA SRS detector has a better gantry angle dependency relative to SRS MapCHECK detector, with a small dependence of less than 1% deviation when the gantry angle varied within ± 120°, while SRS MapCHECK showed a random variation relative to the gantry angle, with a maximum difference of −5.3%.^35^

The results of our study shown that detection of 1 mm outward deviations in MLC was challenging with gamma analysis criteria of (3%/3 mm), (3%/2 mm), and (2%/2 mm) and even an increase in the pass rate was observed when 1 mm MLC deviation occurred (Table 1, Figure 6). However, the average GPR of (3%/1 mm) criterion decreased from 90.4% in the original plan (no deviation) to 81.4% and 62.9% for 1 and 3 mm MLC inward deviations, with average decreases of approximately 9.0% and 27.5%, respectively. These results suggested that a more rigorous criterion was required to detect the similar MLC errors in our study using this special device.

Due to the high positional accuracy requirement of SBRT, sensitivity to setup error (positioning error) over clinic tolerance is essential for QA measurement. We evaluated the sensitivity of the myQA SRS by simulating setup error in three directions. The results showed that the myQA SRS is highly sensitive to positioning error with the (3%/1 mm) GPR, which decreased by averages of 20.9%, 25.7%, and 24.7% for 1 mm setup error in the X, Y, and Z directions, respectively. Meanwhile, the 2 mm DPR decreased by averages of 17.5%, 19.8%, and 17.8% for the same 1 mm set up error in X, Y, and Z directions either. Compared to MLC deviation, the myQA SRS was more sensitive to setup error and capable to detect 1 mm position error even using the GPR of (3%/3 mm) criterion. respectively.

In the report of the AAPM task group No. 218, a criteria of (3%/2 mm) was suggested for global gamma pass rate evaluation in PSQA of IMRT, without a specific recommendations for SRS and SBRT verification but tighter tolerance suggested.^45^ In our end‐to‐end SBRT QA comparison of the myQA SRS device with an EPID dosimetry system, which had high measurement resolution too, the results showed very similar performance in measurement with the two devices. When using a gamma criterion of greater than (2%/2 mm), both the myQA SRS and EPID devices achieved high average GPRs greater than 95%, of which only one case had a GPR of less than 90%, respectively. However, when more stringent (3%/1 mm) criterion was used, the average GPRs of both devices dropped by 5% approximately, with 4 cases had a GPR under 90%, respectively (Table 3). Considering that the requirement for position accuracy (DTA) should be greater than that of dose difference (DD) in SRS and SBRT,^46^ a more stringent DTA should be used for evaluation. For the myQA SRS system and scenarios of our study (3%, 1 mm) is an appropriate and sensitive criterion for gamma pass rate evaluation. The comparison test shows that the myQA SRS device with a detector array of 0.4 mm resolution yielded close results to the high resolution EPID measurement and provided an alternative or complement method of SBRT QA. For example, in the treatment plan of some special cases with eccentric target, the target area cannot be placed at the iso‐center to avoid collision of the tabletop or patient. In this circumstance, the target and the adjacent area may not be included in the measurement range of the EPID, the myQA SRS device can be placed to the corresponding position of the target area for measurement instead.

In SRS and SBRT treatment, the non‐coplanar irradiation technique is often used with rotation in both the couch and gantry. This raises the concern about the couch and gantry combined angle dependency of the dosimetry QA devices, which needs to be carefully inspected and calibrated before their application for non‐coplanar SRS/SBRT QA. It was not included in this work and remaining a further study in the future.

## 5 CONCLUSION

The high‐resolution myQA SRS dosimetry system is able to detect small MLC deviations and setup errors in SBRT plans with high sensitivity. The dose rate dependence should be adequately calibrated before its application and a more stringent GPR (3%/1 mm) evaluation criterion is suggested when it is used for SRS/SBRT QA.

## AUTHOR CONTRIBUTIONS

Manuscript: Yang Zhou, Yimei Liu, Meining Chen, Jun Zhang, Yinglin Peng; Sample acquisition and patient cohort: Jianlan Fang, Liangjie Xiao, Shaomin Huang; Statistical analysis: Zhenyu Qi, Xiaowu Deng. All authors read and approved the final manuscript.

## CONFLICT OF INTEREST STATEMENT

The authors declare no potential conflicts of interest.

## Supporting information

Supporting Information

## Data Availability

The datasets are backed up on the Research Data Deposit (RDD Number: RDDA2023432737, https://www.researchdata.org.cn) and are available upon reasonable request.
