# Supplementary material for: Commissioning and clinical evaluation of a novel high‐resolution quality assurance digital detector array for SRS and SBRT
Source: J Appl Clin Med Phys. 2024 Jan 4;25(4):e14258. doi: 10.1002/acm2.14258 (PMC11005972; doi:10.1002/acm2.14258)
Supplement: Supplementary file 1 — Supporting Information [file ACM2-25-e14258-s001.docx]

**Supplementary Material**

b

a


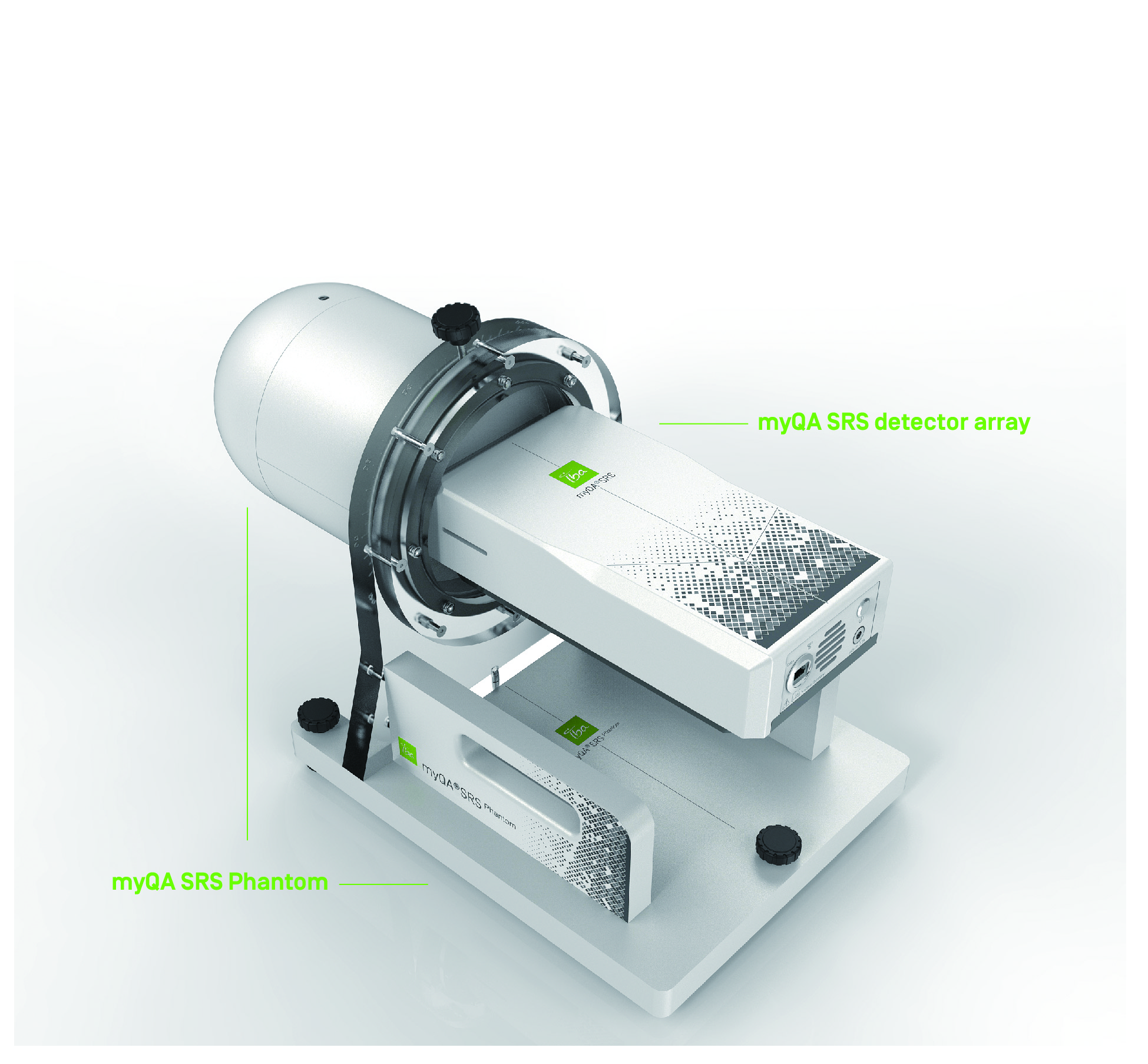

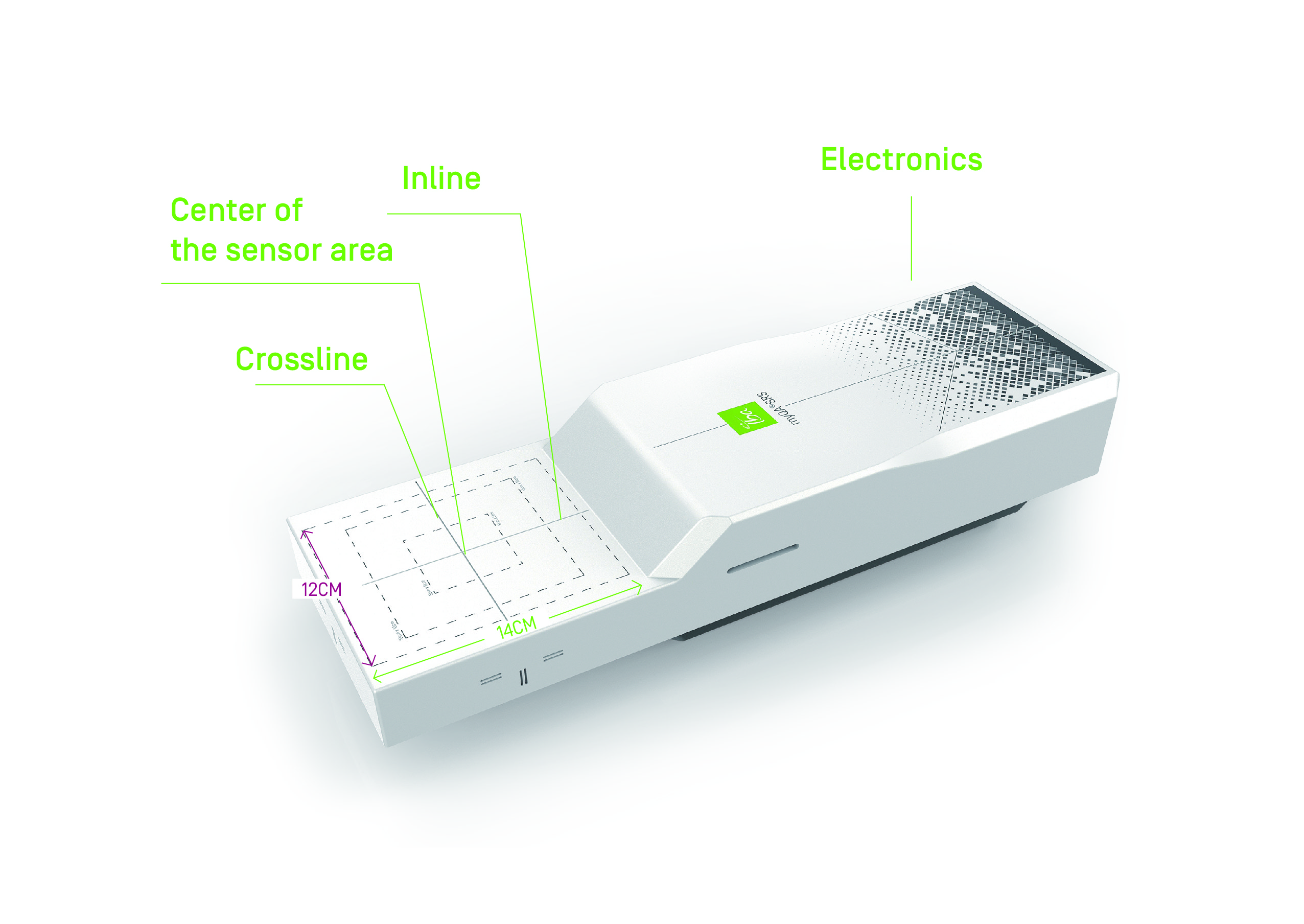


Figure S1. Schematic presentation of the myQA SRS detector: (a) detection platform and (b) sensor.
